# Myosin X is recruited to nascent focal adhesions at the leading edge and induces multi-cycle filopodial elongation

**DOI:** 10.1038/s41598-017-06147-6

**Published:** 2017-10-20

**Authors:** Kangmin He, Tsuyoshi Sakai, Yoshikazu Tsukasaki, Tomonobu M. Watanabe, Mitsuo Ikebe

**Affiliations:** 1 Institute of Vascular Medicine, Peking University Third Hospital and Academy for Advanced Interdisciplinary Studies, Peking University, Key Laboratory of Cardiovascular Molecular Biology and Regulatory Peptides, Ministry of Health, Key Laboratory of Molecular Cardiovascular Sciences, Ministry of Education and Beijing Key Laboratory of Cardiovascular Receptors Research, Beijing, 100191 China; 2 Graduate School of Frontier Biosciences, Osaka University, Osaka, 5650871 Japan; 3 Department of Cellular and Molecular Biology, University of Texas Health Science Center at Tyler, Tyler, TX75708 USA; 4 Laboratory for Comprehensive Bioimaging, RIKEN Quantitative Biology Center (QBiC), Osaka, 5650874 Japan; 5 World Premier International Research Center Initiative, iFReC, Osaka University, Osaka, 5650871 Japan; 6 Department of Pharmacology, University of Illinois Chicago College of Medicine, Boston Children’s Hospital, Chicago, Illinois 60612, USA; 7 Present Address: Department of Cell Biology, Harvard Medical School, and Cellular and Molecular Medicine Program, Boston Children’s Hospital, Boston, MA 02115 USA

## Abstract

Filopodia protrude from the leading edge of cells and play important roles in cell motility. Here we report the mechanism of myosin X (encoded by Myo10)-induced multi-cycle filopodia extension. We found that actin, Arp2/3, vinculin and integrin-β first accumulated at the cell’s leading edge. Myosin X was then gathered at these sites, gradually clustered by lateral movement, and subsequently initiated filopodia formation. During filopodia extension, we found the translocation of Arp2/3 and integrin-β along filopodia. Arp2/3 and integrin-β then became localized at the tip of filopodia, from where myosin X initiated the second extension of filopodia with a change in extension direction, thus producing long filopodia. Elimination of integrin-β, Arp2/3 and vinculin by siRNA significantly attenuated the myosin-X-induced long filopodia formation. We propose the following mechanism. Myosin X accumulates at nascent focal adhesions at the cell’s leading edge, where myosin X promotes actin convergence to create the base of filopodia. Then myosin X moves to the filopodia tip and attracts integrin-β and Arp2/3 for further actin nucleation. The tip-located myosin X then initiates the second cycle of filopodia elongation to produce the long filopodia.

## Introduction

Filopodia are finger-like cell surface projections composed of parallel bundles of actin filaments and are found in a variety of cell types and play sensory or exploratory functions in cell migration, adhesion to the extracellular matrix, guidance towards chemoattractants, neuronal growth-cone path finding and embryonic development^1–3^. Several actin-regulatory proteins, such as CDC42, RIF, Arp2/3 complex, WASP/WAVE, Dia2, Ena/VASP, myosin X, fascin, IRSp53, LPR1, integrin-β and PtdIns(3,4,5)*P*_3_, have been found to regulate filopodia formation and dynamics^4–12^. For most filopodia-related proteins, however, their roles and spatiotemporal dynamics during filopodia formation are still not well understood. For certain proteins, such as Arp2/3 and VASP, contradictory findings have been reported^13–17^. Two alternative models, the convergent elongation model and De novo filament elongation model, emphasizing two different actin filament nucleators, Arp2/3 complex and Dia2, have been proposed for filopodia initiation^1,2,18^.

Myosin X exhibits a characteristic localization at the tip of filopodia^19^. Myosin X overexpression led to a dramatic increase in the number and length of filopodia^20,21^. On the other hand, knockdown of the expression of endogenous myosin X by small interference RNA led to the loss of filopodia^5,8^. These findings suggest that myosin X plays a critical role in filopodia formation. Myosin X is composed of a conserved motor domain in its N-terminal region, a neck region consisting of three IQ motifs that serve as light chain binding sites, a predicted coiled-coil domain and a unique tail domain^22^. The tail contains a PEST domain, three pleckstrin homology domains, a myosin tail homology 4 (MyTH4) domain and a band 4.1/ezrin/radixin/moesin (FERM) domain^23^. It was found that the tail domain could bind to specific cargo molecules such as VASP and β-integrin, although it is not directly shown whether or not myosin X actually transport these proteins^4,21^.

Up to now, the underlying mechanism of myosin X-induced filopodia formation is still poorly understood. A critical finding was that forced dimerization of the tailless myosin X induced filopodia^21^, indicating that myosin X’s motor activity is sufficient to initiate filopodia. On the other hand, full-length myosin X is monomeric^7^. Therefore, a critical question is how myosin X forms a dimer, which is necessary for filopodia formation. Recently, it was found that PtdIns(3,4,5)P_3_ binding at the PH domain of myosin X tail induced the dimerization of myosin X. It was postulated that myosin X binds to PtdIns(3,4,5)P_3_ at the cell’s leading edge to form a dimer, and utilizes its motor function to induce actin filament convergence to initiate filopodia formation^7^.

Since the filopodia induced by the tailless myosin X dimer was short and unstable^5^, it was thought that transportation of the tail-binding cargo molecules played a role in producing the stable and long filopodia. Supporting this view, actin-regulating proteins (e.g. VASP and integrin-β) have been found to be involved in regulating myosin X-induced filopodia formation^4,21^. Myosin X has been shown to bind to and co-localize with Mena/VASP at filopodial tips^21^. The lack of Ena/VASP proteins in cortical neurons markedly inhibited filopodia formation, which could be rescued by the expression of myosin X^24^. It was also reported that myosin X binds to integrin-β, which is involved in filopodia formation^4^. The filopodia protrusion thus is performed by machinery composed of the interactions among actin-associated proteins mediated by myosin X.

The aim of this study is to clarify the mechanism of myosin X-induced filopodia initiation and elongation. With triple-color total internal reflection fluorescence microscopy (TIRFM), we analyzed the spatiotemporal localization of myosin X and actin-regulatory proteins during the initiation and elongation of filopodia. Based upon our observations, we propose a new model of myosin X-induced filopodia formation and phased extension.

## Results

### Spatial distribution of myosin X with actin and actin-regulating proteins along myosin X-induced filopodia

**Figure 1:**
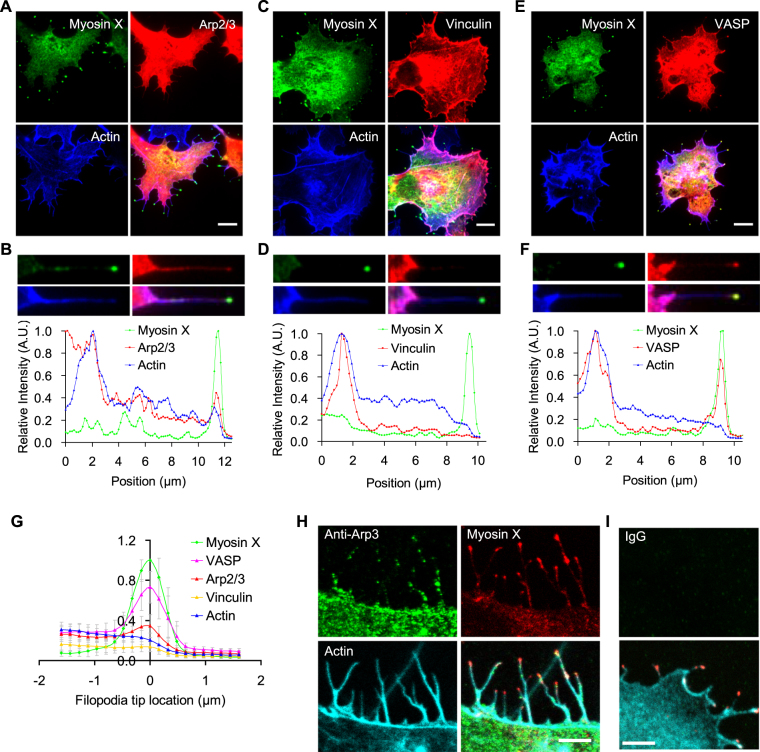
Spatial distribution of myosin X, actin, Arp2/3, vinculin and VASP along myosin X-induced filopodia. COS7 cells were co-transfected with EGFP-myosin X and mCherry-Arp2/3 (**A**), EGFP-myosin X and vinculin-tomato (**C**), or EGFP-myosin X and mCherry-VASP (**E**), and then stained with Alexa Fluor 633-conjugated phalloidin to label actin structures. The cells were imaged using triple-color TIRFM. (**B**,**D** and **F**) The enlarged representative filopodia and the normalized fluorescence intensities of the indicated proteins along filopodia were shown. A small fraction of myosin X was undergoing active intrafilopodial movement toward filopodial tips, which is reflected by the local variation of the intensity traces. The intensity variation depends on the relative amount/number of intrafilopodial EGFP-myosin X in different filopodia. Scale bars, 10 μm. (**G**) Referring to the fluorescence center of EGFP-myosin X at filopodial tips, the normalized fluorescence traces of EGFP-myosin X (n = 17), vinculin-tomato (n = 22), mCherry-Arp2/3 (n = 17), mCherry-VASP (n = 12) and actin (n = 18) from 4–8 cells were aligned, averaged and then overlaid, respectively. (**H**) Localization of endogenous Arp2/3 in myosin X-induced filopodia. The mCherry-myosin X expressing cells were stained with anti-Arp3 antibody followed by Alexa-Fluor 488-conjugated secondary antibody. Actin was stained with Alexa Fluor 633-conjugated phalloidin. (**I**) To show the specificity of Arp2/3 staining, the mCherry-myosin X expressing cells were stained with non-immune IgG (the same concentration as anti-Arp3) followed by Alexa-Fluor 488-conjugated secondary antibody. The upper panel shows the staining of the IgG channel. The lower panel is the merged image of mCherry-myosin X and actin. Scale bars, 5 μm.

Filopodia formation involves reorganization of actin structures, which is regulated by several actin-binding proteins. To study the mechanism underlying myosin X-induced actin rearrangement and filopodia formation, we first investigated the spatial localization of actin-binding proteins along with myosin X. EGFP-myosin X were transiently co-expressed in COS7 cells together with mCherry-Arp2/3, vinculin-tomato, or mCherry-VASP. Then the cells were stained with Alexa Fluor 633-conjugated phalloidin to label actin structures (Fig. 1A–F). With triple-color TIRFM imaging, we found that mCherry-Arp2/3 colocalized with EGFP-myosin X at the tip as well as shaft of filopodia (Fig. 1A and B). By staining the cells using the anti-Arp3 antibody, we confirmed that the endogenous Arp2/3 was localized at both the base and tip of filopodia (Fig. 1H and I). In addition, Arp2/3 was also found at discrete points along the shaft of filopodia (Fig. 1H and I).

In the cells co-expressing EGFP-myosin X and vinculin-tomato, vinculin was found mainly around the bases of filopodia and focal adhesion like structures, but not at filopodial tips (Fig. 1C, and D, and Supplementary Fig. S1). On the other hand, consistent with our previous result^21^, mCherry-VASP showed strong co-localization with EGFP-myosin X at the tip of myosin X-induced filopodia (Fig. 1E and F, and Supplementary Fig. S1).

To more quantitatively analyze the spatial distribution of myosin X, actin, Arp2/3, vinculin and VASP along filopodia, we aligned the normalized fluorescence traces of these proteins according to the fluorescence center of EGFP-myosin X at filopodial tips (Supplementary Fig. S2). Then we averaged the intensity of several aligned fluorescence traces of myosin X, actin, Arp2/3, vinculin and VASP, respectively, and determined the localization and accumulation of these proteins in the tip complex (Fig. 1G). A large amount of VASP along with myosin X accumulated at the tip of myosin X-induced filopodia. Arp2/3 was also found at filopodial tips. Nevertheless, vinculin and actin did not accumulate at filopodial tips.

### The spatiotemporal dynamics of myosin X with actin and actin-regulating proteins during myosin X-induced filopodia formation

**Figure 2:**
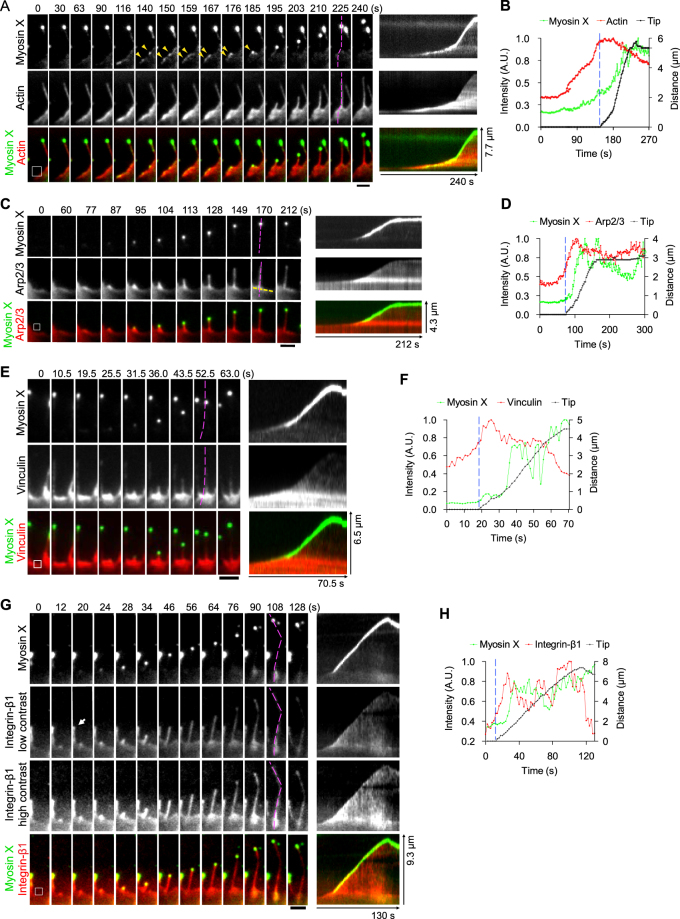
Spatiotemporal dynamics of myosin X, actin and actin-regulating proteins during myosin X-induced filopodia formation. Time-lapse montages showed the dynamics of EGFP-myosin X and mCherry-actin (**A**), EGFP-myosin X and mCherry-Arp2/3 (**C**), EGFP-myosin X and vinculin-tomato (**E**), EGFP-myosin X and integrin-β1-mCherry (**G**) during filopodia formation imaged using dual-color TIRFM. The corresponding kymographs of these proteins along filopodial extension (purple dashed lines) are shown. The yellow arrows in (**A**) show the lateral movement and then fusion of two EGFP-myosin X puncta during filopodia formation. (**B**,**D**,**F** and **H**) The fluorescence intensity of EGFP-myosin X at the base of newly formed filopodia (mean fluorescence of the region-of-interest (ROI) boxes in **A**, **C**, **E** and **G**; before the blue dashed lines) and at the filopodial tip during elongation (after the blue dashed lines), together with the fluorescence intensity of mCherry-actin (**B**), mCherry-Arp2/3 (**D**), vinculin-tomato (**F**) and integrin-β1-mCherry (**H**) at the base of filopodia (mean fluorescence of the same ROI boxes in **A**,**C**,**E** and **G**) during filopodia formation are shown. ROI boxes are shown in the first images of each time lapse series (**A**,**C**,**E**, and **G**). The ROI boxes at the filopodia initiation sites are used for direct comparison between the accumulation rates of myosin X and other proteins during filopodia initiation. The time course of the change in fluorescent intensity of proteins at the site of filopodia initiation was monitored and compared with the time course of myosin X accumulation at the tip of filopodia during filopodia elongation. This was done after the blue dashed line in the fluorescent traces in (**B**,**D**,**F**, and **H**). The tip position of filopodia was determined by the fluorescence center of EGFP-myosin X. The arrow in (**G**) points to a pre-existing filopodia with integrin-β1 and myosin X localized at the tip. Scale bars, 2 μm.

To clarify the mechanism of myosin X-induced filopodia formation, we monitored the spatiotemporal dynamics of myosin X along with actin or actin-regulating proteins during filopodia formation. As shown in Fig. 2A, the gradual increase of mCherry-actin was observed at discrete points of the cell’s leading edge, which suggests a local actin polymerization. After actin accumulation at these points, myosin X started to move laterally along the leading edge of the polymerized actin. The laterally moved myosin X accumulated to form a clump, where the clustering of actin and the subsequent filopodia initiation took place (Fig. 2A and Supplementary Video 1). Interestingly, we found that the accumulation of actin at the base of filopodia preceded the accumulation of myosin X (Fig. 2B).

Simultaneous imaging of EGFP-myosin X and mCherry-Arp2/3 revealed that during filopodia initiation, the local accumulation of Arp2/3 occurred slightly earlier than that of myosin X at the cell’s leading edge (Fig. 2C and D, and Supplementary Video 2). During filopodia extension, Arp2/3 was localized throughout the newly formed filopodia. A small fraction of Arp2/3 was translocated toward and eventually accumulated at the tip of the growing filopodia (Fig. 2C). This suggests that myosin X does not carry Arp2/3 directly. Instead, Arp2/3 is translocated to filopodial tip after myosin X accumulation and then helps to create the tip complex.

Vinculin was also gathered at the leading edge prior to myosin X accumulation, from where nascent filopodia protruded. However, vinculin was not promptly translocated to the tip of the newly formed filopodia (Fig. 2E and Supplementary Video 3). It should be noted that the accumulation of vinculin at the base of nascent filopodia preceded the accumulation of myosin X (Fig. 2F).

Integrin-β1-mCherry also accumulated at discrete locations along the cell’s leading edge prior to the accumulation of myosin X. During filopodia extension, while EGFP-myosin X was translocated to the tip of filopodia, integrin-β1-mCherry was distributed throughout the nascent filopodia and gradually accumulated at the filopodial tip (Fig. 2G and Supplementary Video 4). On the other hand, consistent with the previous report^4^, integrin-β1 was also found at the tip of pre-existing filopodia along with myosin X (Fig. 2G, arrow). Thus, myosin X moved to the filopodial tip first and then integrin-β1 slowly concentrated. The results indicate the fast dissociation of integrin-β1 from myosin X, and integrin-β1 does not stay with myosin X during its movement. Since integrin-β has a low affinity with the C-terminal FERM domain of myosin X^4^, presumably by diffusion, integrin-β1 is gradually accumulated at the filopodia tip where myosin X localizes. In other words, we propose that rather than direct transportation, myosin X functions as a sink for integrin-β1 localization at filopodial tips.

### Convergence of the structural complex composed of actin and actin-regulating proteins at the cell periphery by myosin X

**Figure 3:**
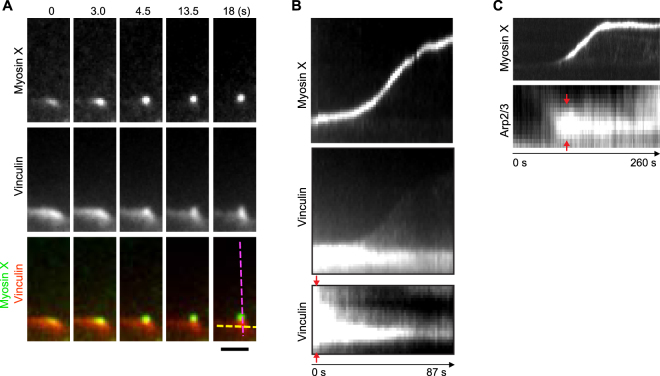
Myosin X converges local-nucleated actin-regulating protein complex during filopodia initiation. (**A**) Time-lapse montages of EGFP-myosin X and vinculin-tomato showed the convergence of local-accumulated vinculin structure during filopodia initiation. (**B**) The corresponding kymographs (top and middle panels) of myosin X and vinculin along filopodial extension (purple dashed line in **A**) show the dynamic changes of myosin X and vinculin during filopodia initiation and extension. The bottom kymograph shows the convergence of vinculin at the root of filopodia perpendicular to the direction of filopodia extension (yellow dashed line in **A**). (**C**) Kymographs of myosin X along filopodial extension (purple dashed line in Fig. 2C) and Arp2/3 at the root of filopodia perpendicular to the direction of filopodia extension (yellow dashed line in Fig. 2C) are shown for the time-lapse movie shown in Fig. 2C. The red arrows in (**B**) and (**C**) point to the vinculin or Arp2/3 initially localized with significant width along the leading edge of cells from where nascent filopodia extended. Scale bar, 2 μm.

To further investigate the spatiotemporal dynamics of the actin-binding protein complex at the cell periphery during filopodia initiation, we tracked the convergence of vinculin and Arp2/3 at nascent focal adhesion along with myosin X (Fig. 3). Initially, the locally accumulated vinculin and Arp2/3 localized at the cell’s leading edge with significant width. Then they started to narrow when myosin X was gathered, from where filopodia was subsequently initiated (Figs 2C and 3A, Supplementary Fig. S3A). By recording the kymograph of vinculin or Arp2/3 at the root of myosin X-induced filopodia, we found that the actin-binding protein complex at the cell periphery was converged during myosin X-induced filopodia initiation and elongation (Fig. 3B and C, Supplementary Fig. S3B), similar to that of actin structures during filopodia initiation (Fig. 2A). During filopodia elongation, while myosin X moved toward the tip of filopodia, vinculin predominantly stayed at the root of filopodia (Figs 2E and 3A).

### Effects of knockdown of actin-regulating proteins on myosin X-induced filopodia formation

**Figure 4:**
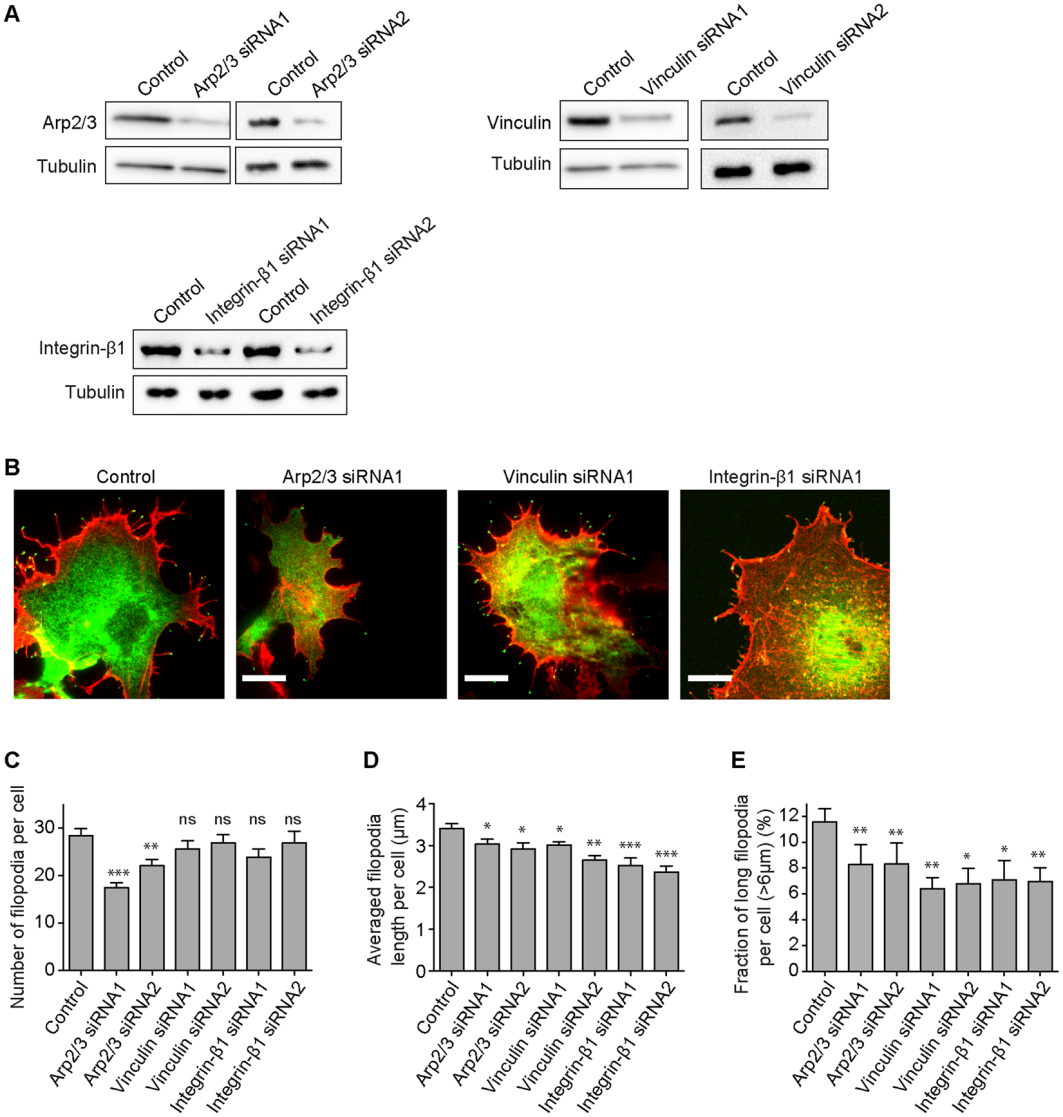
Effect of knockdown of integrin-β1 and other actin-regulating proteins on myosin X-induced filopodia formation. (**A**) Western blotting of COS7 cells transfected with control, Arp2/3, vinculin or integrin-β1 siRNA, respectively. To exclude the non-specific effect, two siRNA sequences were used for each protein. Tubulin was used as loading control. Full-length blots were presented in Supplementary Fig. S6. (**B**) COS7 cells expressing EGFP-myosin X (green) were transfected with control, Arp2/3, vinculin or integrin-β1 siRNA, respectively. Then the cells were stained with Alexa Fluor 633-conjugated phalloidin (red) and imaged using TIRFM. (**C**) The average filopodia number ( ± s.e.m.), (**D**) the average filopodia length ( ± s.e.m.), and (**E**) the relative frequency ( ± s.e.m.) of the long filopodia ( > 6 μm) per control, Arp2/3, vinculin or integrin-β1 siRNA treated cell were quantified (Control, n = 2331 from 82 cells; Arp2/3 siRNA1, n = 818 from 47 cells; Arp2/3 siRNA2, n = 621 from 30 cells; Vinculin siRNA1, n = 1099 from 43 cells; Vinculin siRNA2, n = 723 from 30 cells; Integrin-β1 siRNA1, n = 1017 from 40 cells; Integrin-β1 siRNA2, n = 911 from 34 cells). Asterisks above columns indicate the statistical significance versus the control siRNA treated cells using the Mann–Whitney test (*P < 0.05, **P < 0.01, ***P < 0.001). Scale bars, 10 μm.

Previously we found that elimination of the C-terminal FERM domain of myosin X diminished the length of myosin X-induced filopodia, while the number of filopodia did not change markedly^25^. Since the FERM domain interacts with integrin-β, we examined the effect of knockdown of integrin-β1 and another two actin-associated proteins, Arp2/3 and vinculin, on filopodia length. siRNA treatment suppressed the expression of Arp2/3, vinculin and integrin-β1, respectively (Fig. 4A). Knockdown of the expression of integrin-β1 decreased filopodia length (Fig. 4D) and effectively reduced the fraction of long filopodia ( > 6 μm) (Fig. 4E). Nevertheless, the formation of filopodia was not apparently affected (Fig. 4C). This result is consistent with the previous report^25^ and suggests that the interaction of myosin X with integrin-β is important for the production of long filopodia. We also found that knockdown of the expression of Arp2/3 and vinculin decreased the number of long filopodia (Fig. 4C–E). These results suggest that integrin-β, Arp2/3 and vinculin are involved in filopodia elongation. On the other hand, the number of filopodia was notably influenced neither by the siRNA for integrin-β1 nor by the siRNA for vinculin (Fig. 4C).

### Directional changes during filopodia extension

**Figure 5:**
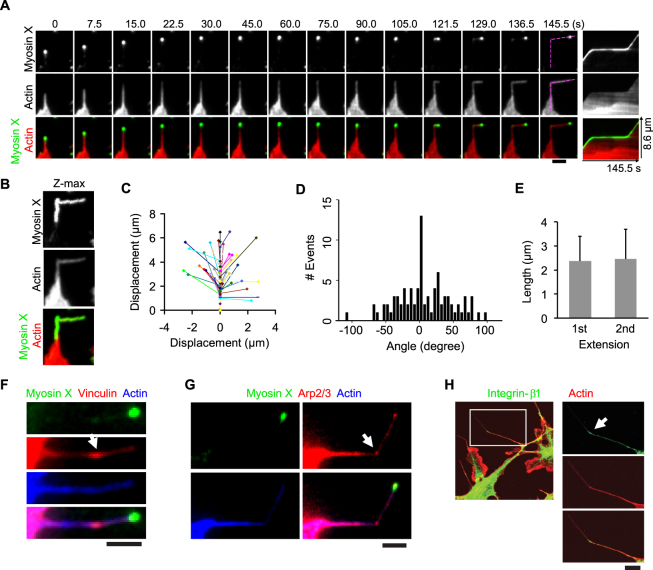
The multi-cycle extension and directional changes during filopodia extension. (**A**) Time-lapse montages of EGFP-myosin X and mCherry-actin show a directional change during the multi-cycle extension of filopodia. The corresponding kymographs of myosin X and actin along filopodial extension (purple dashed lines) are shown. The maximum intensity projections of EGFP-myosin X and mCherry-actin are shown in (**B)**. (**C**) The representative line traces show the displacements and directional changes during filopodia extension, with the three nodes for each line representing a filopodia’s initiation, length at the end of the first extension, and position at the end of the second extension. (**D**) Histograms of the angle changes between the first and second cycle of filopodia extension (n = 85). (**E**) Average length (± s.d.) of the filopodial extension during the first and second cycle of extension (n = 85). (**F** and **G**) Local accumulation of vinculin-tomato and mCherry-Arp2/3 at sites where directional changes occured (arrows). (**H**) Localization of integrin-β1 in myosin X-induced filopodia. The cells expressing myc-myosin X were stained with antibodies against integrin-β1 followed by the Alexa Fluor 488-conjugated secondary antibody. Actin was stained using the Alexa Fluor 633-conjugated phalloidin. The arrow points to the local accumulation of integrin-β1 at the site where the directional change occurred. Scale bars, 2 μm.

Next, we asked why knockdown of integrin-β1 and vinculin expression decreased the formation of long filopodia. To address this question, we monitored the dynamic filopodia elongation process in cells co-expressing EGFP-myosin X and mCherry-actin using dual-color live-cell imaging. As shown in Fig. 5A and Supplementary Video 5, right after the forward movement during filopodia extension, the filopodial tip-located myosin X slowed down its movement. After the retraction of the filopodia tip, another phase of filopodia extension was launched as revealed by the fast forward movement of the tip-located myosin X. During the multi-cycle filopodia extension, we noticed a sharp directional change between the first and the second phases of filopodia extension (Fig. 5A). The multi-cycle filopodia extension was also observed in the cells co-expressing myosin X with vinculin or Arp2/3 (Supplementary Fig. S4). By creating the maximum intensity projection of EGFP-myosin X and mCherry-actin at each spatial location during filopodia extension, we can clearly show the trajectory of the filopodia and the sharp directional changes of both myosin X and actin (Fig. 5B). Using this method, we measured the angles during directional changes (Fig. 5C and D) and the lengths of both the first and second extensions (Fig. 5E). We found that filopodia showed both left- and right-hand directional changes during the multi-cycle elongation (Fig. 5C). The angle changes were widely distributed for both left- and right-hand changes (Fig. 5D). The lengths during the first and second phase of filopodia protrusion were practically the same (2.37 ± 1.02 μm and 2.45 ± 1.24 μm for the first and the second extension, respectively) (Fig. 5E). Interestingly, we found the accumulation of vinculin-tomato, mCherry-Arp2/3 and endogenous integrin-β1 at the sites where directional changes occurred (Fig. 5F–H). The accumulation of vinculin-tomato and mCherry-Arp2/3 at the base of the second filopodia extension was also observed (Supplementary Fig. S5). These results suggest that the second filopodia extension initiates from the nascent focal adhesion at the filopodial tip where integrin-β1, Arp2/3 and vinculin are present.

### Myosin X moves constantly to the tip of filopodia

**Figure 6:**
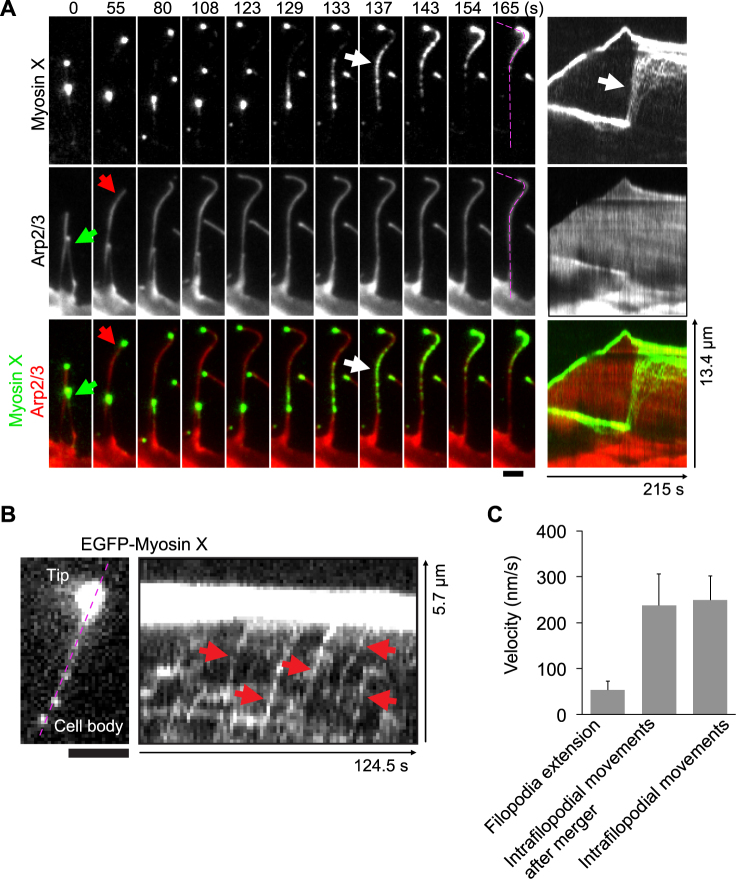
Myosin X behavior during merger of filopodia. (**A**) Time-lapse montages of EGFP-myosin X and mCherry-Arp2/3 show the quick movement of myosin X (white arrow) originally located at the tip of the short filopodia (green arrows) toward the tip of the longer filopodia (red arrows). Kymographs of EGFP-myosin X and mCherry-Arp2/3 along filopodial extension (purple dashed lines) were obtained, with the white arrow pointing to the quick movement of myosin X to the tip of the longer filopodia. (**B**) The kymograph of EGFP-myosin X movement within filopodia (purple dashed line) shows the fast intrafilopodial movements of myosin X (red arrows) from the cell body to the tip of a pre-existing filopodia. (**C**) Average velocities ( ± s.d.) of the forward movements of filopodial tips during filopodia extension (Filopodia extension, n = 58), the quick forward intrafilopodia movements of myosin X after filopodial fusion as shown in (**A**) (Intrafilopodial movements after merger, n = 47) and the intrafilopodial movements of myosin X as shown in (**B**) (Intrafilopodial movements, n = 55). Scale bars, 2 μm.

After extension for several micrometers, filopodia elongation slowed down and the tip-located myosin X also ceased movement. As shown in Fig. 6A and Supplementary Video 6, when a short filopodia met and fused with an adjacent longer filopodia, the tip-located myosin X in the short filopodia restarted a fast forward movement toward the tip of the longer filopodia, as illustrated in kymographs (Fig. 6A). This movement was much faster than filopodia extension (Fig. 6C). Interestingly, no further protrusion was observed for the longer filopodia when nearly all myosin X moved to the tip of the longer filopodia.

The quick movement of the tip-located myosin X was similar to the intrafilopodial single-molecule movement of myosin X (Fig. 6B). By measuring the velocities from the recorded kymographs using a method described previously^25^, we found that at room temperature, the intrafilopodial single-molecule movement of myosin X (247 ± 68 nm/s, Fig. 6B) and the quick forward movement of myosin X within the long filopodia after filopodia merging (237 ± 69 nm/s, Fig. 6A) showed similar velocities, both of which were much faster than the rate of filopodia extension (53 ± 19 nm/s) (Fig. 6C). This result suggests that the fast movement represents myosin X moving along the existing actin bundles in the longer filopodia. Arp2/3 localized at the tip of the shorter filopodia gradually redistributed throughout the longer filopodia and accumulated at the tip (Fig. 6A).

## Discussion

To initiate filopodia formation, at least 10 actin filaments are required to overcome the membrane tension^26^. This raises a key issue on the origin of the bundled actin filaments: whether they arise from lamellipodia or form independently of the latter^3^. A convergent elongation model was proposed, in which filopodial bundles were produced by reorganization of the lamellipodial dendritic network^2^. However, these reorganized actin bundles did not protrude much from the leading edge of lamellipodia (such bundles were called Λ-precursors) to form the typical long and thin filopodia structure. Moreover, lamellipodia could advance along filopodia to embed the filopodia actin bundles into the lamellipodia actin network^27,28^. Thus, it is unclear whether the bundled actin filaments of a typical filopodia are produced from lamellipodia. In this study, we attempt to clarify the mechanism of myosin X-induced filopodia formation by analyzing the real-time dynamics of myosin X together with actin-regulatory proteins.

We found an intense nucleation event of actin along the cell’s leading edge prior to filopodia formation. Moreover, local nucleation of Arp2/3 was also observed, from where subsequent filopodia formation took place. The finding suggests that there is an abundance of short, branched actin filaments before filopodia formation. Notably, actin and Arp2/3 nucleation were launched from a relatively broad area along the cell’s leading edge instead of a single point. We found that along with actin and Arp2/3, the focal adhesion proteins integrin-β1 and vinculin were also accumulated at discrete points along the cell’s leading edge prior to myosin X accumulation, from where nascent filopodia extended. On the other hand, knockdown of the expression of these proteins did not influence the number but significantly shortened the length of filopodia. The results suggest that these proteins influence either the stability or the second cycle filopodia extension. We previously found the lateral movement of myosin X along the leading edge of nucleation sites^5^. The gradual clustering of myosin X was accompanied by the convergence of the local nucleated actin and Arp2/3 structure, which was observed after the nucleation of actin and Arp2/3.

Once myosin X is accumulated at the nascent focal adhesion, filopodia formation is induced. A critical finding is that myosin X induces multi-cycle filopodia extension, and the second phase of filopodia extension changes the direction with a wide-angle distribution. Consistently, we found that the long filopodia showed a bend at the middle of entire filopodia. Interestingly, the focal adhesion proteins vinculin and integrin-β1 were localized at the bend. Moreover, Arp2/3 was also found at the bend, suggesting that actin nucleation for new actin filaments takes place at this site. It has been thought that Arp2/3 is present at lamellipodia to produce branched actin structure. In the present study, we found that Arp2/3 was also present in filopodia, suggesting that the branched actin structure is possibly present if Arp2/3 is activated, which requires further study.

The present results suggest that the second phase of filopodia extension requires focal adhesion and the associated polymerized actin structure induced by Arp2/3. Myosin X converges the branched actin structure to parallel bundles to induce the second phase of filopodial extension. Supporting this view, we also observed the gradual accumulation of integrin-β1 and Arp2/3 at the myosin X-induced filopodia tips. Consistently, knockdown of Arp2/3, integrin-β1 or vinculin reduced the number of long filopodia. As cells rely on the finger-like filopodia to probe and interact with their surroundings especially in processes such as axon guidance and angiogenesis^19^, the directional changes during phased extension may play an important role in the path-finding of cells.

Interestingly, when the tip-located myosin X encountered another longer filopodia, it restarted a quick movement toward the tip of the longer filopodia, with a velocity similar to that of the intrafilopodial myosin X movement. It should be noted that the observed velocity of myosin X movement was slower than that reported previously^25,29^, which is likely due to the difference in the imaging temperature (i.e., room temperature in the present study while 37 °C in previous studies). Moreover, in order to perform the long-term live-cell tracking (~9–10 min) while minimizing photobleaching, we imaged the cells at a time interval of 1.5 s, which was much longer than that used in previous studies^25,29^, which may also result in the slower velocity. The newly accumulated myosin X, however, did not readily induce a second phase of extension of the newly merged thicker filopodia. This suggests that myosin X accumulation at the filopodia tip is not sufficient to induce the second cycle of filopodia elongation. It is also possible that there is a limit to the number of actin filaments in the newly formed filopodia, determined by the crosslinking properties of fascin or the number of Dia2 molecule available. We also observed that Arp2/3 was gradually translocated to the filopodia tip after myosin X accumulation. Moreover, we found that Arp2/3 and vinculin accumulated at the tip prior to the initiation of the second cycle of filopodia elongation. Based upon these findings, we think that the subsequent translocation of Arp2/3 along with vinculin and integrin-β1 to the filopodial tip is important to produce actin nucleation for the second cycle of filopodia extension. Knockdown of Arp2/3, vinculin and integrin-β1 by siRNA reduced the length of myosin X-induced filopodia, which further supports that the focal adhesion and local accumulation of these proteins are important for myosin X-induced filopodia formation. On the other hand, siRNA induced down-regulation of these proteins did not notably reduced the number of filopodia. This suggests that the decrease in the expression of these proteins does not diminish the first phase of filopodia formation. Since the accumulation of these proteins at the base of nascent filopodia seems to be much larger than the subsequent phase, it is plausible that the reduced amount of these proteins can be sufficient for the first cycle of filopodial elongation.

It was reported that deletion of the FERM domain of myosin X, a integrin-β binding domain, decreased the length but not number of filopodia^25^. Consistent with this finding, we find that the binding of myosin-X with integrin-β1 is involved in the formation of long filopodia, probably *via* the multi-cycle elongation. The question is how myosin X without FERM domain can initiate the first cycle extension of filopodia. We think that accumulation of myosin X at nascent focal adhesion creates adequate force for actin convergence, which is critical for filopodia initiation. It is plausible that myosin X without the FERM domain can concentrate at the nascent focal adhesion at the cell periphery through the binding with other focal adhesion components. Alternatively, myosin X without the FERM domain may have a weak affinity for integrin-β, which is sufficient for myosin X attraction due to a high concentration of integrin-β. Another possibility is the recruitment of myosin X at nascent focal adhesion by actin, which accumulates at the nascent focal adhesion prior to the recruitment of myosin X. Since both myosin X and its tailless forced dimer bind F-actin, this may explain how the tailless dimer can induce short filopodia^5^. Further studies are required to clarify this question.

It was found that myosin X is monomeric and forms a dimer when phospholipids bind to its PH domain^5,7^. And it was proposed that myosin X exists in an inactive monomeric conformation in cells before reaching the cell periphery, at which phospholipids bind to and activate myosin X and induce the formation of myosin X dimers at the root of filopodia^5,7^. In the present study, we observed that myosin X moved along the cell periphery and accumulated at the nascent focal adhesion. It is plausible that myosin X encounters PtdIns(3,4,5)*P*_3_ at the cell periphery and starts to move along the leading edge toward the nascent focal adhesion, where the myosin X binding partners are localized. The two-headed myosin X can bind to two adjacent single actin filaments^30–32^. By acting like a zip fastener, myosin X helps to converge the local nucleated actin filaments to bundles.

Based upon these findings, we propose the following model. Myosin X is recruited to the nascent focal adhesion at the cell’s leading edge. Myosin X moves laterally along the leading edge, converges the actin structure, and then produces the base of filopodia. Then myosin X moves on the parallel bundles of actin filaments in the newly produced filopodia and accumulates at the filopodial tip. Once myosin X is accumulated at the filopodial tip, integrin-β diffuses along the filopodia and gathers at the tip via binding to myosin X. Arp2/3 is also recruited to facilitate actin nucleation. Myosin X again converges the actin structures to facilitate actin bundle formation and subsequently induces the second cycle of filopodia extension.

## Materials and Methods

### Plasmids

Bovine myosin X cDNA was kindly provided by Dr. David Corey (Harvard University). EGFP-myosin X was constructed as described previously^21^. The cDNAs of human actin related protein 2/3 complex subunit 3 (Arp3) isoform 1 (NCBI Reference Sequence: NM_001278556.1), human actin β (NCBI Reference Sequence: NM_001101.3) and human VASP (NCBI Reference Sequence: NM_003370.3) were inserted into the vector mCherry-C1 to generate the plasmids mCherry-Arp2/3, mCherry-actin and mCherry-VASP. The cDNA of human integrin subunit β1 (integrin-β1) isoform 1 A (NCBI Reference Sequence: NM_002211.3) was inserted into the vector mCherry-N1 to generate the plasmid integrin-β1-mCherry. Vinculin-tomato and vinculin-EGFP (Mus musculus vinculin; NCBI Reference Sequence: NM_009502.4) were kind gifts from Dr. Yasushi Okada (RIKEN Quantitative Biology Center, Osaka, Japan).

### Cell culture and transfection

African green monkey kidney COS7 cells (American Type Culture Collection) were cultured at 37 °C and 5% CO_2_ in Dulbecco’s modified Eagle’s medium supplemented with 10% fetal calf serum. Cells growing in a Collagen I (BD Bioscience) coated 35-mm glass-bottom dish were transfected with FuGENE HD transfection reagent (Promega) according to the manufacturer’s instructions. Twenty four hours after transfection, the cells were used for experiments.

### siRNA experiments

COS7 cells seeded at 30% confluency were transfected with the following siRNA: Control siRNA (mismatched control duplexes, Ambion); Arp2/3 siRNA1 (5′-AAAUCCUAAUGGAGACAAA-3′, Ambion), Arp2/3 siRNA2 (5′-CAUCACGGUUGGAACGAGAACUUAA-3′, Ambion); Vinculin siRNA1 (5′-GCUUCAAUCAAAAUUCGAA-3′, Ambion), Vinculin siRNA2 (5′-GGCUGCGGUUGGUACUGCUAAUAAA-3′, Ambion); integrin-β1 siRNA1 (5′-CCGUAGCAAAGGAACAGCA-3′, Ambion), integrin-β1 siRNA2 (5′-CCUAAGUCAGCAGUAGGAACAUUAU-3′, Ambion). siRNA transfection was performed at a final concentration of 75 nM using siPORT Amine transfection agent (Ambion) according to the manufacturer’s instructions. The efficiency of silencing was assessed at 3 days after transfection by western blot using antibodies against Arp2 (N1C3, 1:2000 dilution of 1 mg/ml stock; Genetex), integrin-β1 (12G10, 1:1000 dilution of 1 mg/ml stock; abcam), vinculin (hVIN-1, 1:2000 dilution of 1 mg/ml stock; Sigma) or β-tubulin (T4026, 1:5000 dilution of 2.6 mg/ml stock; Sigma).

### Western blot analysis

Proteins separated by SDS-PAGE were transferred to a nitrocellulose membrane (Bio-Rad) in a tank apparatus at 70 V for 30 min. The membrane was blocked with 5% nonfat dry milk, 0.05% Tween 20 in Tris-buffered saline (TBS) for 1 h. The membrane was incubated with the primary antibodies, followed by the horseradish peroxidase-conjugated secondary antibodies (BioRad) in 0.15% BSA in PBS. Detection was performed using Super Signal West Pico (Pierce Chemical).

### Live-cell imaging

The objective-type TIRFM used an inverted Nikon Eclipse Ti microscope equipped with a Nikon Apo objective (100X/1.49NA) and an electron multiplier type charge-coupled device camera (EMCCD, Andor iXonX3). The laser excitation used in this experiment was 488 nm (Melles Griot) to excite EGFP and 561 nm (Coherent Sapphire) to excite mCherry and tomato. Time-lapse images were obtained by exiting the sample by 488 nm (~7–8 mW) and 561 nm lasers (~0.3–0.4 mW) alternatively and imaged by an EMCCD. The time interval between the two lasers excitation was 1.5 s. The fluorescence of EGFP or mCherry was selectively collected with the corresponding bandpass filters (FF01–520/35-25 or FF02-641/75-25, Semrock) with the exposure time of 100 ms. The cells were imaged at room temperature (~25 °C) in the medium without phenol red (Gibco). Images analysis and single particles tracking were performed with Image J (NIH). Kymographs were generated using the plugin in Image J with the line width of 3 pixels.

### Immunofluorescence

The plasmids or siRNA transfected cells were fixed with fix buffer (4% formaldehyde in PBS) at room temperature for 20 min, washed twice with PBS, and permeabilized with 0.5% Triton X-100 in PBS for 10 min at room temperature. After a brief wash with PBS, the cells were incubated with the blocking buffer (3% BSA in PBS) for 1 h at room temperature. Then the cells were incubated with the diluted anti-integrin-β1 antibody (4B7R, 1:400 dilution of 0.2 mg/ml stock; abcam) or anti-Arp2 antibody (N1C3, 1: 400 dilution of 1 mg/ml stock; Genetex) in blocking buffer for 1 h at room temperature. After 3 times wash with PBS, the cells were incubated with the diluted Alexa Fluor 488-conjugated secondary antibodies (1:500 dilution of 0.2 mg/ml stock; Invitrogen) in blocking buffer for 1 h at room temperature. For actin staining, cells were incubated with Alexa Fluor 567 or 633-conjugated phalloidin (1:500 dilution of 6.6 μg/ml stock; Invitrogen) for 30 min at room temperature. After 3 times wash with PBS, the samples were imaged with the same TIRFM system as mentioned above. For triple-color TIRFM imaging, the samples were excited sequentially by a 633 nm laser, a 488 nm laser and a 561 nm laser, and imaged by an EMCCD.

## Electronic supplementary material

Supplementary information

Spatiotemporal dynamics of myosin X and actin during myosin X-induced filopodia formation

Spatiotemporal dynamics of myosin X and Arp2/3 during myosin X-induced filopodia formation

Spatiotemporal dynamics of myosin X and vinculin during myosin X-induced filopodia formation

Spatiotemporal dynamics of myosin X and integrin-β during myosin X-induced filopodia formation

The multi-cycle extension and directional changes during filopodia extension

Myosin X moved constantly to the tips of filopodia
