# Supplementary material for: Myosin X is recruited to nascent focal adhesions at the leading edge and induces multi-cycle filopodial elongation
Source: Sci Rep. 2017 Oct 20;7:13685. doi: 10.1038/s41598-017-06147-6 (PMC5651867; doi:10.1038/s41598-017-06147-6)
Supplement: Supplementary file 1 — Supplementary information [file 41598_2017_6147_MOESM1_ESM.pdf]

Myosin X is recruited to nascent focal adhesions at the leading edge and induces multi-cycle filopodial elongation

Kangmin He[1,2,6,#], Tsuyoshi Sakai[3,#], Yoshikazu Tsukasaki[3,4], Tomonobu M. Watanabe[2,4,5]*, Mitsuo Ikebe[3]*

[1] Institute of Vascular Medicine, Peking University Third Hospital and Academy for Advanced Interdisciplinary Studies, Peking University, Key Laboratory of Cardiovascular Molecular Biology and Regulatory Peptides, Ministry of Health, Key Laboratory of Molecular Cardiovascular Sciences, Ministry of Education and Beijing Key Laboratory of Cardiovascular Receptors Research, Beijing 100191, China

[2] Graduate School of Frontier Biosciences, Osaka University, Osaka 5650871, Japan

[3] Department of Cellular and Molecular Biology, University of Texas Health Science Center at Tyler, TX75708, USA.

[4] Laboratory for Comprehensive Bioimaging, RIKEN Quantitative Biology Center (QBiC), Osaka 5650874, Japan

[5] World Premier International Research Center Initiative, iFReC, Osaka University, Osaka 5650871, Japan

[6] Present address: Department of Cell Biology, Harvard Medical School, and Cellular and Molecular Medicine Program, Boston Children's Hospital, Boston, MA 02115, USA

[#] The authors contributed equally to this work.

*Correspondence to:

Mitsuo Ikebe, Department of Cellular and Molecular Biology, University of Texas Health Science Center at Tyler, TX75708, USA, E-mail: mitsuo.Ikebe@umassmed.edu

or

Tomonobu Watanabe, Laboratory for Comprehensive Bioimaging, Quantitative Biology Center, Riken, Suita, JAPAN 565-0874, E-mail: tomowatanabe@riken.jp

Supplementary Information

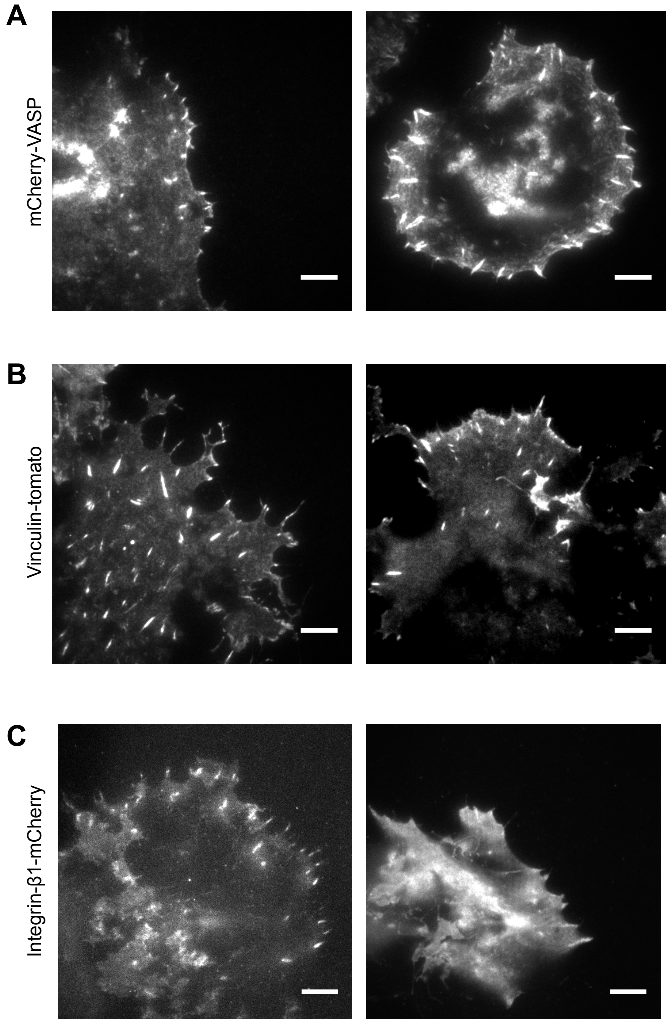

Supplementary Figure S1. Localization of transiently expressed mCherry-VASP, vinculin-tomato and integrin-β1-mCherry in COS7 cells. The cells were imaged using TIRFM and two cells were shown for each construct. Scale bars, 10 μm.

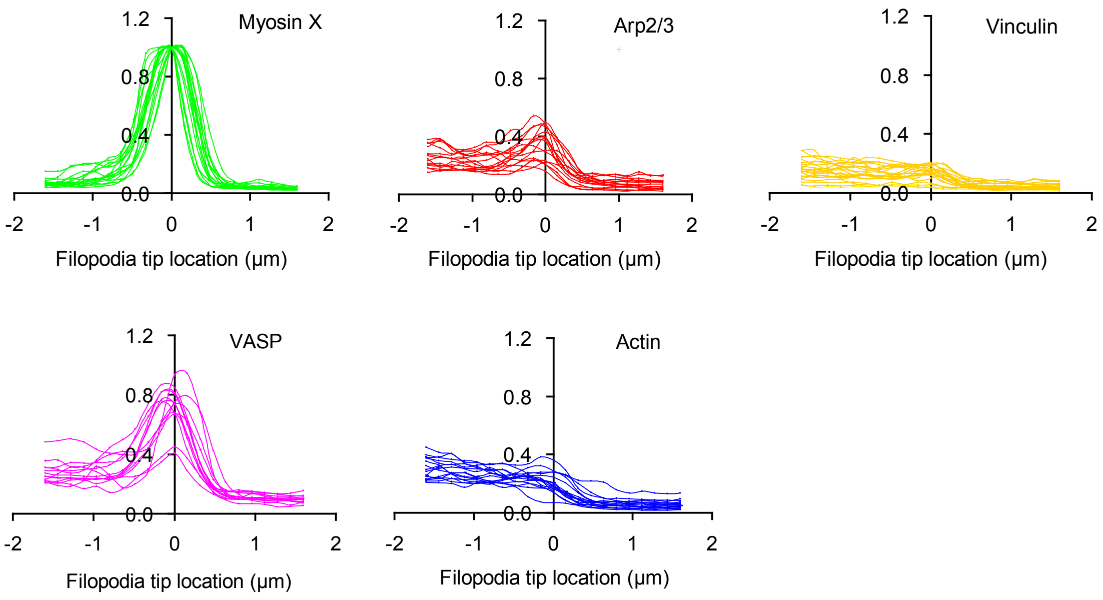

Supplementary Figure S2. Distribution of myosin X, actin, Arp2/3, vinculin and VASP at the tips of myosin X-induced filopodia. COS7 cells were cotransfected with GFP-myosin X and mCherry-Arp2/3, GFP-myosin X and vinculin-tomato, or GFP-myosin X and mCherry-VASP, and then stained with Alexa Fluor 633-conjugated phalloidin to label actin structure. The cells were imaged using triple-color TIRFM. Referring to the fluorescence center of GFP-myosin X at filopodial tips, the normalized fluorescence traces of GFP-myosin X (n = 17), vinculin-tomato (n = 22), mCherry-Arp2/3 (n = 17), mCherry-VASP (n = 12) and actin (n = 18) from 4-8 cells were aligned, respectively, to generate the averaged figures as shown in Fig. 1G.

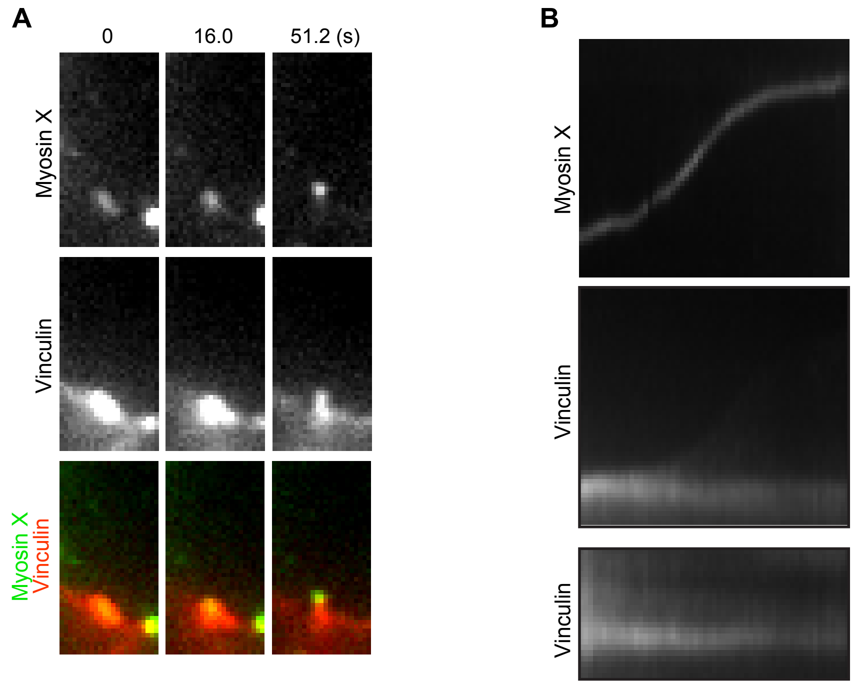

Supplementary Figure S3. Re-arrangement/convergene of local-nucleated vinculin during myosin X-induced filopodia initiation. (A) Time-lapse montages of GFP-myosin X and vinculin-tomato showed the convergence of local-accumulated vinculin structure during filopodia initiation imaged using dual-color TIRFM. (B) The kymographs with display intensity ranging from 0 to 20000 of the 16-bit images were shown for the kymographs in Figure 3B.

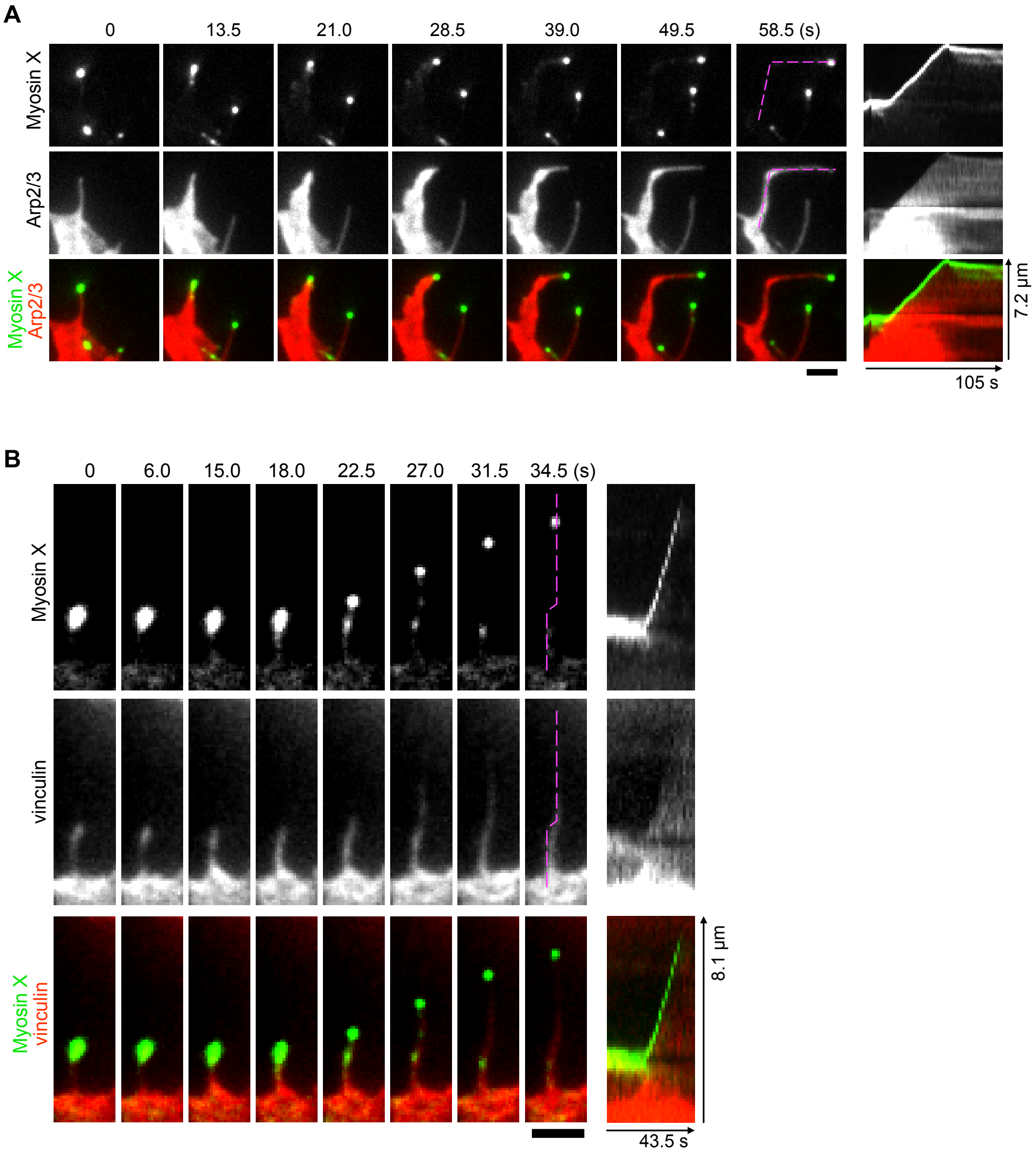

Supplementary Figure S4. The multi-cycle extension and directional changes during myosin X-induced filopodia extension. (**A**) Time-lapse montages of GFP-myosin X and mCherry-Arp2/3 showed the multi-cycle extension and directional change during filopodia elongation. The kymographs of myosin X and Arp2/3 along filopodial extension (purple dashed lines) are shown. (**B**) Time-lapse montage of GFP-myosin X and vinculin-tomato showed the multi-cycle extension and directional change during filopodia elongation. The kymographs of myosin X and vinculin along filopodial extension (purple dashed lines) are shown. Scale bars, 2 µm.

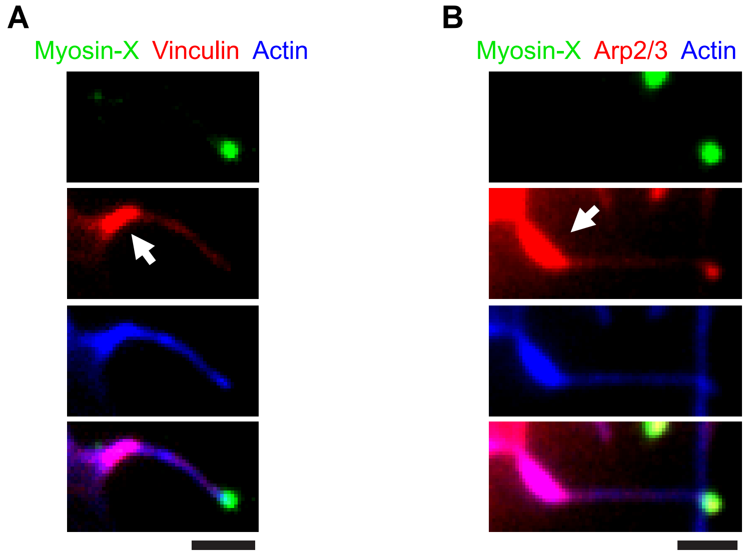

Supplementary Figure S5. Local accumulation of vinculin and Arp2/3 at the root of the second filopodia extension. The cells were cotransfected with GFP-myosin X and vinculin-tomato (**A**), or GFP-myosin X and mCherry-Arp2/3 (**B**) and then stained with Alexa Fluor 633-conjugated phalloidin to label actin structures. Then the cells were imaged using triple-color TIRFM. Arrows point to the accumulation of vinculin and Arp2/3 at the root of the second filopodia extension. Scale bars, 2 μm.

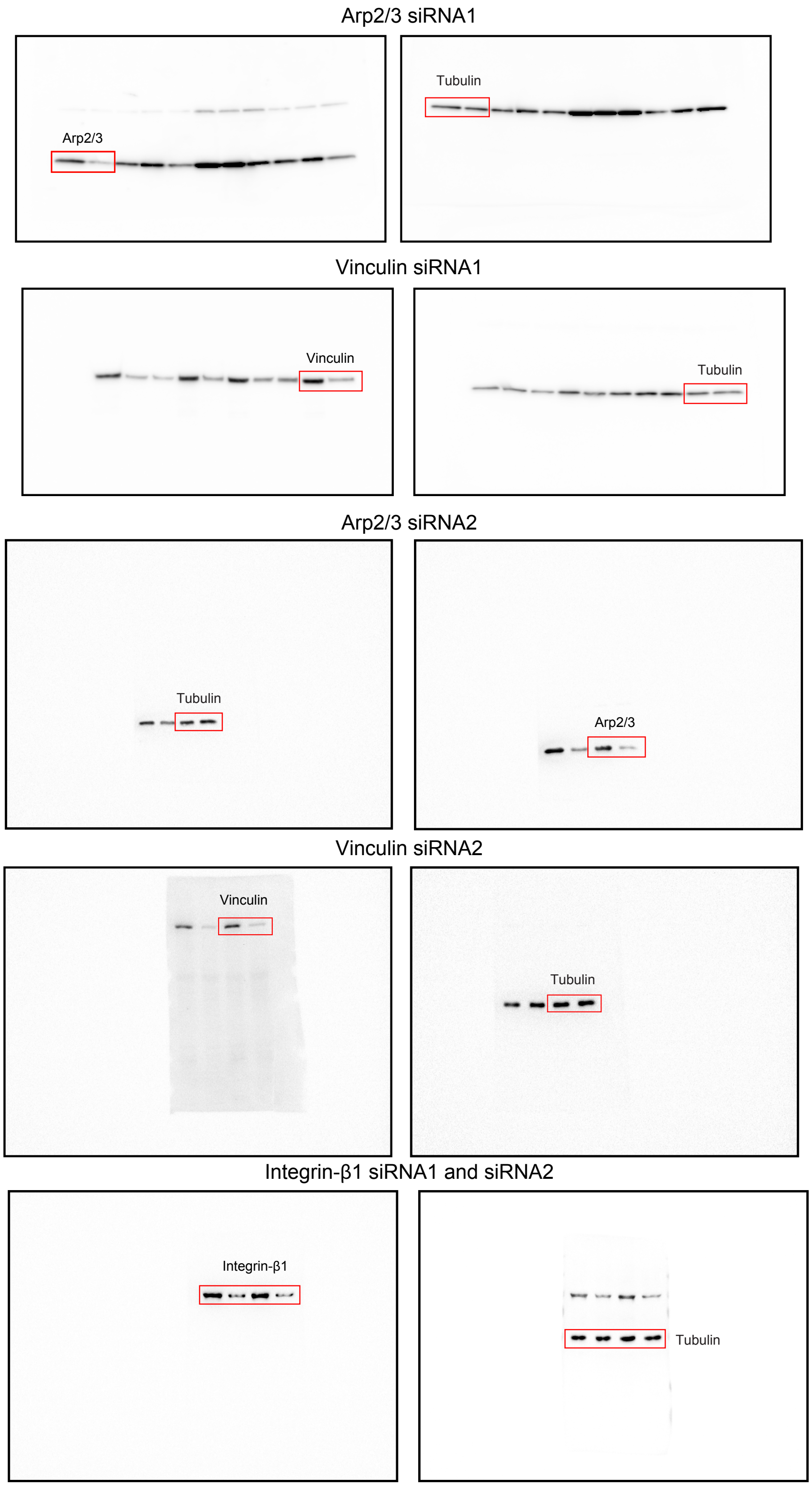

Supplementary Figure S6. Full-length blots for Figure 4A.

Supplementary Video Legends

Supplementary Video 1. Spatiotemporal dynamics of myosin X and actin during myosin X-induced filopodia formation. The GFP-myosin X and mCherry-actin expressing cell was imaged using dual-color TIRFM.

Supplementary Video 2. Spatiotemporal dynamics of myosin X and Arp2/3 during myosin X-induced filopodia formation. The GFP-myosin X and mCherry-Arp2/3 expressing cell was imaged using dual-color TIRFM.

Supplementary Video 3. Spatiotemporal dynamics of myosin X and vinculin during myosin X-induced filopodia formation. The GFP-myosin X and vinculin-tomato expressing cell was imaged using dual-color TIRFM.

Supplementary Video 4. Spatiotemporal dynamics of myosin X and integrin-β during myosin X-induced filopodia formation. The GFP-myosin X and integrin-β1-mCherry expressing cell was imaged using dual-color TIRFM.

Supplementary Video 5. The multi-cycle extension and directional changes during filopodia extension. The GFP-myosin X and mCherry-actin expressing cell was imaged using dual-color TIRFM.

Supplementary Video 6. Myosin X moved constantly to the tips of filopodia. The GFP-myosin X and mCherry-Arp2/3 expressing cell was imaged using dual-color TIRFM. After the lower short filopodia met and fused with an adjacent longer filopodia, the tip located myosin X in the short filopodia restarted a fast forward movement toward the tip of the longer filopodia,
